# Diversity and composition of soil bacteria between abandoned and selective-farming farmlands in an antimony mining area

**DOI:** 10.3389/fmicb.2022.953624

**Published:** 2022-07-22

**Authors:** Renyan Duan, Yihuan Du, Zhiwei Chen, Yaqi Zhang, Wei Hu, Li Yang, Guohong Xiang, Yucai Luo

**Affiliations:** College of Agriculture and Biotechnology, Hunan University of Humanities, Science and Technology, Loudi, China

**Keywords:** soil pollution, agricultural practices, bacteria, 16S rRNA, heavy metal

## Abstract

**Background and aims:**

Land abandonment and selective farming are two common management methods to restore the soil conditions of low-pollution farmland in mining areas. The soil bacterial community plays an important role in farmland soil restoration; however, few studies have compared the composition and diversity of soil bacteria between the abandoned farmlands (AFS) and selective-farming farmlands (FFS). Here, the effects of AFS and FFS on soil properties and bacterial diversity were evaluated in an antimony (Sb) mining area in southern China. This study aimed to identify effective land management methods in terms of positive or negative changes in soil environment and bacterial diversity.

**Methods:**

16S rRNA high-throughput sequencing was used to compare the diversity and composition of soil bacteria between AFS and FFS in the Xikuangshan (the largest Sb mine in the world).

**Results:**

Compared to AFS, FFS had higher Sb concentration and nutritional properties (e.g., available N, P, and K) and lower Zn concentration (*p* < 0.05). The bacterial alpha diversity including Chao1 index, Simpson index, Shannon index and Pielou_−_e index in FFS was higher than AFS (*p* < 0.05). At the phylum level, FFS had higher relative abundances of *Chloroflexi*, *Acidobacteria*, *Gemmatimonadetes*, and *Rokubacteria*, and lower relative abundances of *Firmicutes*, *Actinobacteria*, and *Bacteroidetes*. At the genus level, FFS had higher relative abundances of *Acidothermus*, *Haliangium*, and *Rokubacteriales*, and lower relative abundances of *Bacillus*, *Rhodococcus*, *Sphingomonas*, and *67-14*. Redundancy analysis indicated that soil heavy metal content and soil fertility were closely correlated with the soil bacterial community. Altogether, selective farming of low-pollution farmland in the mining area can improve soil properties and soil bacterial diversity.

## Highlights

- Selective farming of farmland can improve soil nutritional properties.
- Selective farming significantly improved the bacterial alpha diversity.
- Selective farming changed the composition and abundances of soil dominant bacteria.

## Introduction

Due to many environmental problems related to heavy metal soil pollution, concern about the soil quality of farmlands near metal mining areas is increasing (Tang et al., 2020; Castellano-Hinojosa et al., 2021; Han et al., 2022; Khmelevtsova et al., 2022). Farmlands with heavy metal pollution can show low productivity and poor safety of crops, resulting in the agricultural production activities stopped (Deng et al., 2015). However, to meet the production and living needs of local residents, it is unrealistic to abandon large areas of polluted farmland (especially low-pollution farmland). To address this problem, different strategies including metal removal, selective farming and safe farming with agronomic measures have been explored in the past decades (Li, 2019; Jin et al., 2020; Chen et al., 2021; Deng et al., 2021; Han et al., 2022; Khmelevtsova et al., 2022). Among these, selective farming on low-pollution farmland is one of the common farming methods for some countries (including China) with large populations and less cultivated lands (Hou et al., 2018; Han et al., 2022; Khmelevtsova et al., 2022).

Farming of farmlands can affect soil physicochemical properties and biological processes (García-Orenes et al., 2010; Wang et al., 2019; Khmelevtsova et al., 2022). Previous studies have shown that farmland planting can destroy the size of soil aggregates and increase soil porosity, thus changing the stability of soil structure (Deng et al., 2015). Farmland planting can also affect the activity, biomass and diversity of soil microbe, and change the content, valence and stability of heavy metals in soil (Deng et al., 2015; Huang et al., 2020). These changes can reduce or increase the degree of pollution by heavy metals in soil, thereby affecting microbial and biochemical processes in farm ecosystems (Raiesi, 2006). In addition, the health of cultivated land is closely related to soil bacterial characteristics (Zhou and Ding, 2007; Wang et al., 2019; Khmelevtsova et al., 2022). Soil bacterial diversity can be used to evaluate the soil quality of contaminated soil, as it can reflect the effects of farming methods, nitrogen input and land restoration (Jusselme et al., 2013; Nivelle et al., 2016). Therefore, the composition and diversity of soil bacteria can be used as an essential indicator to evaluate cultivated land restoration.

There is little information on the impact of land abandonment and selective farming on soil properties and soil bacteria in mining areas, although these are typical management methods in China (Sun et al., 2018; Bai et al., 2021). It is assumed that selective farming and abandonment will lead to different soil characteristics and soil bacteria. In this study, we sampled the soil of local selective-farming farmlands and abandoned farmlands for high-throughput sequencing of bacteria and detected soil properties (heavy metal content and nutrient status). We compared the differences in soil bacterial composition and diversity between the two farming methods to provide a basis for follow-up research on the remediation of heavy metal polluted farmlands from the perspective of bacteria.

## Materials and methods

### Experimental site and sampling

The study sites were located in the Sb mining area (Xikuangshan, the largest Sb mine in the world) of Hunan Province, China (Supplementary Figure S1). Mining activities gradually stopped in the 1990s. According to previous research, there are different degrees of combined pollution of Sb, As, Cd, Pb, Hg, and Zn in the surrounding farmland soil (Zhou et al., 2019; Bai et al., 2021).

A preliminary investigation and visit to the farmlands around the mining area showed although the pollution conditions of these farmland were similar, due to the large number of manual relocation in the mining area, some farmlands close to the residence was selectively planted with sweet potato (*Ipomoea batatas* L.) and maize (*Zea mays* L.) for 10–15 years, while some farmlands far away were abandoned for 10–15 years. In the study area, selective-farming land was cultivated, fertilized in the form of complex fertilizer (NPK) and irrigated in spring/summer, and the annual application rates of N, P and K were about 310–360, 120–140, 230–270 kg/ha, respectively. In these abandoned farmlands, the dominant plants were *Boehmeria nivea*, *Pteridium aquilinum* var. *Latiusculum*, *Miscanthus floridulus*, and *Erigeron annuus*. Maize (Guo et al., 2011; Wang et al., 2016) and sweet potato (Huang et al., 2020) are cropped in low-pollution farmland as their low accumulation of heavy metals. Planting maize and sweet potato instead of rice in low-pollution areas of heavy metals are one of the measures to protect human health and the agricultural economy (Wang et al., 2016; Huang et al., 2020).

### The collection of farmland soil and determination of soil properties

To study the impact of different farming methods on farmland restoration in mining areas, five abandoned farmlands (AFS, representing natural restoration) and five farming farmlands (FFS, representing the conventional soil management) were selected randomly to sample farm soils of about 10–15 cm deep. In total, 10 farmlands were sampled. In each farmland, five soil sub-samples were collected and then they were fully mixed to obtain a uniform sample of each site (Sun et al., 2020). Visible coarse roots, stones and soil animals were removed. The soil samples were sieved (2 mm). All collected composite samples were placed in a polyethylene sealed plastic bag, labeled, and carried back to the laboratory in an iced box. Each soil sample was artificially divided into two subgroups. The first subgroup was air-dried at ambient temperature to test the contents of soil nutrients (e.g., available N, P, and K) and heavy metals (e.g., Sb, As, Cd, Pb, Zn, and Hg). The second subset was stored at −80°C for soil bacterial DNA extraction.

The contents of heavy metals (Sb, As, Cd, Pb, Zn, and Hg) were measured by PinAAcle atomic absorption spectrometer (Perkineller Inc., United States). The determination of soil nutrient properties was carried out using the method of Zhang and Fang (2007) and Sun et al. (2020). Briefly, the nitrogen content was determined by micro-kjeldahl digestion. NH^4+^-N and NO^3−^-N in soil were extracted with 2 M KCl (w/v, 1:5), and their sum was the available N (AN) content. After digestion with H_2_SO_4_-HClO_4_ and extraction with 0.5 M NaHCO_3_, the available phosphorus (AP) content was determined by the molybdenum blue colorimetry method. After digestion with HF-HClO_4_ and extraction with 1 M NH_4_OAC, the available potassium (AK) content was measured by flame photometer.

### Illumina MiSeq platform sequencing and sequence analysis

The total bacterial DNA was purified from 0.5 g of each homogenized soil samples using the E.Z.N.A.® Soil DNA Kit (Omega Bio-Tek, United States). The V3-V4 regions of bacterial 16S rRNA gene were amplified and sequenced with 338F (forward primers, 5′-ACTCCTACGGGAGGCAGCAG-3′) and 806R (reverse primers, 5′-GGACTACHVGGGTWTCTAAT-3′; Deng et al., 2020a; Duan et al., 2021). PCR conditions included an initial denaturation step at 98°C for 5 min, followed by 25 cycles including denaturation at 98°C for 15 s, annealing at 55°C for 30 s, extension at 72°C for 30 s, and a final extension at 72°C for 5 min. High-throughput sequencing of 16S rRNA amplicons was performed on the Illumina MiSeq platform at Shanghai Personal Biotechnology Co., Shanghai, China.

### Statistical analysis

The ambiguous sequence readings in the raw 16S rRNA data were discarded by QIIME software (version 1.8.0), and then the high-quality sequences were clustered with 97% similarity. The sequence data visualization and statistical analyses for the soil bacterial community were carried out using the R software package (version 3.2.0) and QIIME software. A Venn diagram was created with R software to visualize the similarity and overlap of OTU numbers between FFS and AFS (Fouts et al., 2012). The Chao1 richness index, Simpson index, Shannon index and Pielou evenness index were used to evaluate the differences of alpha diversity indices. Principal coordinate analysis (PCoA), Non-metric multidimensional scaling analysis (NMDS) and hierarchical cluster analysis were used to quantify the differences and similarities of bacterial beta diversity between samples (Deng et al., 2020a). The correlation between soil parameters and bacterial abundance was studied by redundancy analysis (RDA; Guo et al., 2019). A Kruskal–Wallis test was used to test the statistical differences of bacterial communities between FFS and AFS.

## Results

### Nutrient and heavy metal concentration in soil

Compared to AFS, the contents of heavy metals and soil nutrients in FFS were significantly different (Table 1). Compared to AFS, the concentration of Sb in FFS was significantly higher (*p* < 0.05), while the concentration of Zn was significantly lower (*p* < 0.05), and there was no significant difference in the contents of other heavy metals (e.g., As, Cd, Pb, and Hg; *p* > 0.05; Table 1). Compared to AFS, due to the influence of long-term fertilization in selective-farming farmlands, the soil nutritional status in FFS was significantly improved (*p* < 0.05). The available N, available P, and available K in farmed farmlands improved by 79.96%, 369.68%, and 312.99%, respectively (Table 1).

**Table 1:** Heavy metal and soil nutrients in the FFS and the AFS.

Sample	Sb (mg/kg)	As (mg/kg)	Cd (mg/kg)	Pb (mg/kg)	Hg (mg/kg)	Zn (mg/kg)	Available N (mg/kg)	Available P (mg/kg)	Available K (mg/kg)
AFS	554.40 ± 12.42 b	40.22 ± 4.23 a	1.36 ± 0.04 a	44.76 ± 3.95 a	0.68 ± 0.04 a	118.11 ± 4.72 a	105.80 ± 17.57 b	15.14 ± 1.45 b	15.40 ± 1.67 b
FFS	804.56 ± 17.82 a	38.55 ± 9.87 a	1.30 ± 0.03 a	42.15 ± 4.65 a	0.61 ± 0.03 a	89.68 ± 2.48 b	190.40 ± 12.81 a	71.11 ± 3.66 a	63.60 ± 0.55 a

Data are reported as mean ± SE (*n* = 5). Different letters in the same column indicate significant differences between cultivated and abandoned farmland.

### Alpha diversity index and beta diversity analysis

**Figure 1:**
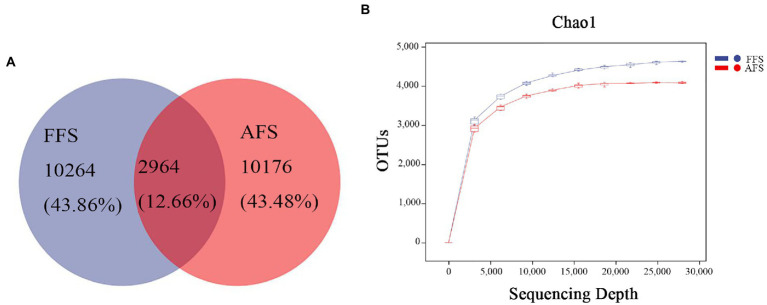
Venn diagrams **(A)** and the sparse curve **(B)** in bacterial communities in the selective-farming farmlands (FFS) and the abandoned farmlands (AFS). Different colored shapes represent different groups.

The number of OTUs identified in FFS and AFS was 13,228 and 13,140, respectively. There were 2,964 overlapping OTUs in the two groups, and 10,264 and 10,176 unique OTUs in the soil from FFS and FFS, respectively (Figure 1A). The sparse curves reached a plateau, indicating that the sequencing depth was sufficient (Figure 1B).

**Figure 2:**
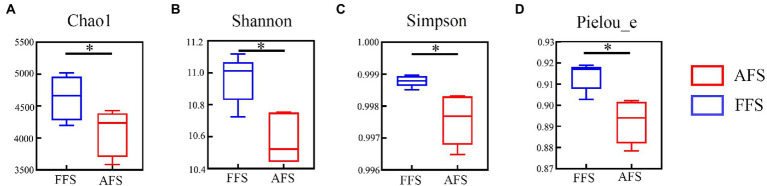
Alpha diversity of soil bacterial communities **(A-D)** in the FFS and the AFS. *p < 0.05, mean ± SD, Kruskal-Wallis test.

Alpha diversity between FFS and AFS was estimated by Chao1 (Figure 2A), Shannon (Figure 2B), Simpson (Figure 2C), and Pielou_−_e indexes (Figure 2D). These indexes in the FFS were significantly higher than in AFS (Kruskal–Wallis tests, *p* < 0.05; Figure 2).

**Figure 3:**
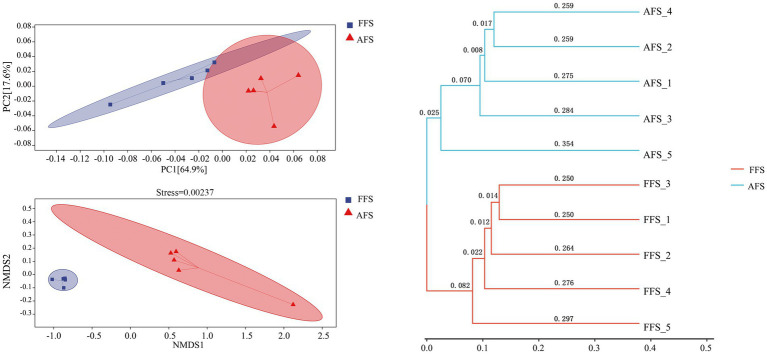
Principal coordinates analysis (PCoA) and non-metric multidimensional scaling analysis (NMDS) based on Bray–Curtis distance represent the differences in the soil bacteria between the FFS and the AFS. The hierarchical cluster analysis shows clusters of bacterial communities based on weighted UniFrac with 100% support at all nodes. Different colored shapes represent different groups.

Beta diversity was assessed to quantify the impact of farming methods on the soil bacterial community structure (Figure 3). The PCoA plots indicated that the soil bacterial communities could be divided into two groups (Figure 3). PCoA plots could explain 64.9% (PC1) and 17.6% (PC2) of the variation in soil bacteria, respectively. NMDS plots showed that there were some inter-group differences (stress = 0.00237), in which FFS and AFS did not overlap, indicating that the soil bacterial community of FFS was significantly different from that of AFS (Figure 3).

### Taxonomic composition analysis

**Figure 4:**
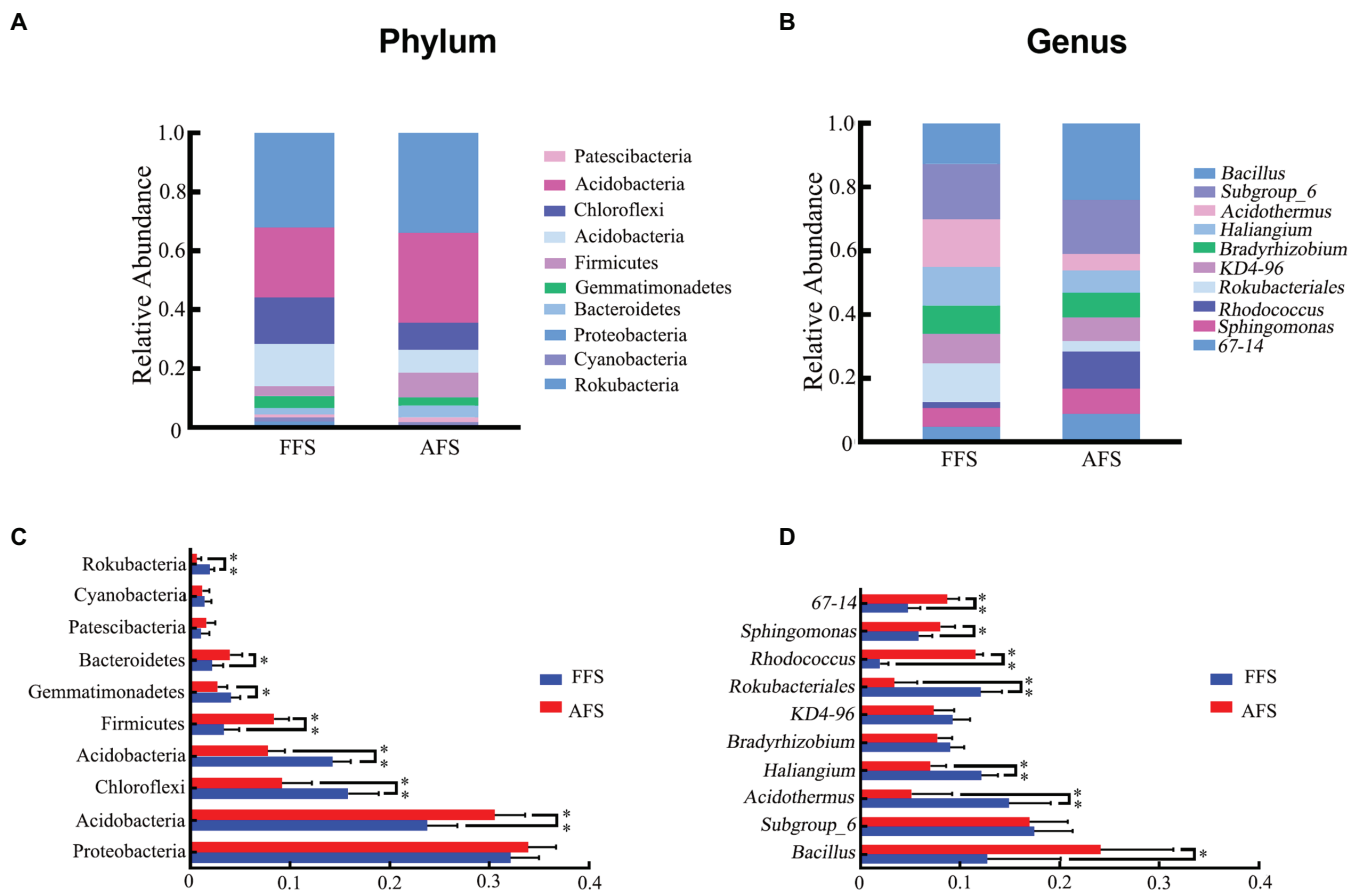
Relative abundance of 10 dominant bacterial phyla **(A, C)** and 10 dominant genera **(B, D)** (relative abundance >1% in at least one sample) in the soil bacteria for the FFS and the AFS (mean ± SD, Kruskal–Wallis test, *p < 0.05, **p < 0.01). Different colored shapes represent different phyla or genus.

To further study the change in soil bacteria between FFS and AFS, we analyzed the compositional changes of soil bacteria in different categories (Figures 4A,B). At the phylum level, 10 domain bacterial phyla (relative abundance >1% in at least one sample) were obtained, including *Proteobacteria*, *Actinobacteria*, *Chloroflexi*, *Acidobacteria*, *Firmicutes*, *Gemmatimonadetes*, *Bacteroidetes*, *Patescibacteria*, *Cyanobacteria*, and *Rokubacteria* (Figure 4A). The dominant phyla with relative abundances >10% were *Proteobacteria* (29.46%–36.75%) and *Actinobacteria* (23.14%–34.66%; Figure 4C). *Proteobacteria*, the most dominant bacteria, showed 33.90% and 32.14% abundances in AFS and FFS, respectively. Notably, the abundance of *Actinobacteria*, *Chloroflexi*, *Acidobacteria*, *Firmicutes*, *Gemmatimonadetes*, *Bacteroidetes,* and *Rokubacteria* showed significant differences between FFS and AFS (Kruskal–Wallis test, *p* < 0.05, Figure 4C). Compared to AFS, the relative abundances of *Actinobacteria*, *Firmicutes*, and *Bacteroidetes* significantly decreased in FFS, while that of *Chloroflexi*, *Acidobacteria*, *Gemmatimonadetes*, and *Rokubacteria* significantly increased in FFS (Kruskal–Wallis test, *p* < 0.05, Figure 4C). The seed network diagram showed that *Actinobacteria* was a node phylum in the networks under FFS and AFS (Supplementary Figure S2).

At the genus level, the top 10 identified genera were *Bacillus*, *Subgroup_6*, *Acidothermus*, *Haliangium*, *Bradyrhizobium*, *KD4-96*, *Rokubacteriales*, *Rhodococcus*, *Sphingomonas*, and *67-14* (Figure 4B). *Bacillus*, *Sphingomonas*, *67-14*, *Acidothermus*, *Haliangium*, *Rokubacteriales*, and *Rhodococcus* showed significant differences in abundance between FFS and AFS (Kruskal–Wallis test, *p* < 0.05, Figure 4D). In addition, the average abundances of *Bacillus*, *Rhodococcus*, *Sphingomonas*, and *67-14* were significantly lower in FFS than in AFS (Kruskal–Wallis test, *p* < 0.05, Figure 4D). On the contrary, the average abundances of *Acidothermus*, *Haliangium*, and *Rokubacteriales* were significantly higher in FFS compared to AFS (Kruskal–Wallis test, *p* < 0.05, Figure 4D).

**Figure 5:**
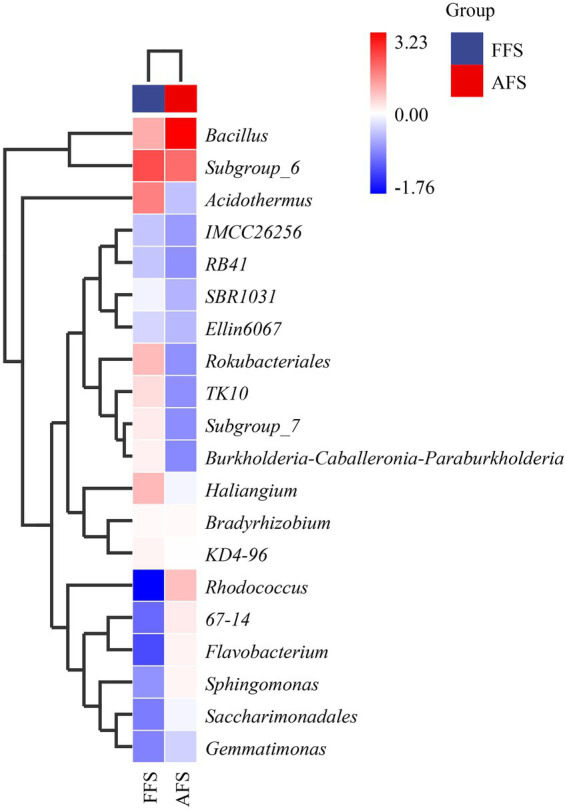
The heatmap of top 20 clusters for species abundance (Genus) in the SFS and the AFS.

To compare the differences in the domain species composition of soil bacteria at the genus level between FFS and AFS, the top 20 genera with average abundances were selected as the analysis objects, and their abundance data were used to draw the genus-level species composition heat map of species clusters (Figure 5). Through horizontal comparison, the top 20 genera in average abundance showed obvious differences between FFS and AFS.

**Figure 6:**
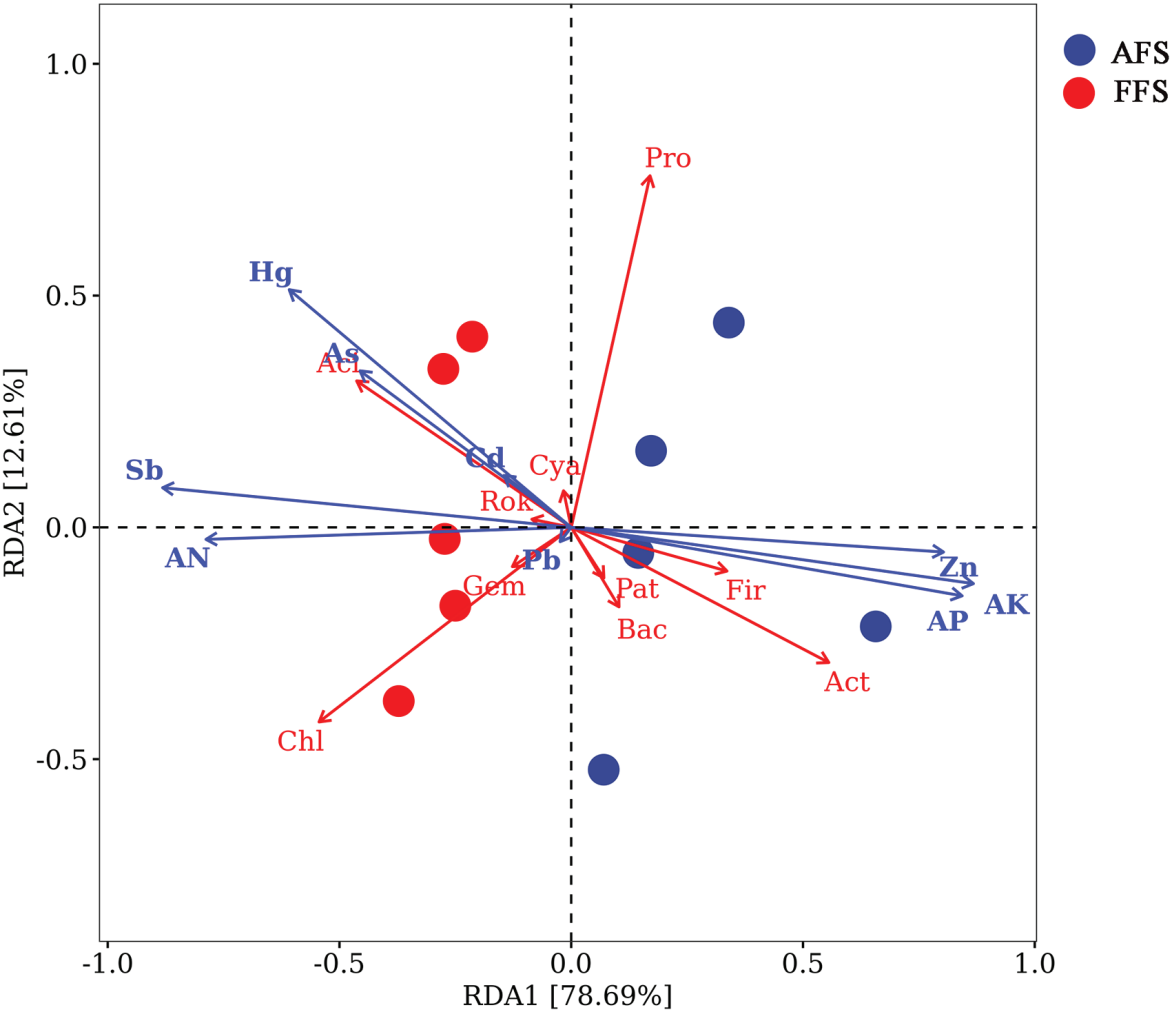
Redundancy analysis (RDA) of bacterial communities using soil properties as environmental parameters in the FFS and the AFS. The different colored points represent the two different types of management. The arrows represent soil properties, including available phosphorus (AP), available nitrogen (AN), and available potassium (AK). The two different circle colors represent FFS and AFS. On the RDA axis, the relationships between environmental variables and the axis were determined by arrow length and angle size. AK, available K; AN, available N; AP, available P; Aci, Acidobacteria; Act, Actinobacteria; Bac, Bacteroidetes; Chl, Chloroflexi; Cya, Cyanobacteria; Fir, Firmicutes; Gem, Gemmatimonadetes; Rok, Rokubacteria; Pat, Patescibacteria; and Pro, Proteobacteria.

The redundancy analysis (RDA) explained 78.69% (RDA1) and 12.61% (RDA2) of the variation, respectively (Figure 6). Figure 6 shows that the heavy metals (Sb, Hg, As, Cd, and Pb) and soil indicators (AN) were positively correlated with *Cyanobacteria*, *Rokubacteria*, *Gemmatimonadetes*, *Acidobacteria,* and *Chloroflexi*. Heavy metal Zn and soil indicators (AK and AP) were positively correlated with *Patescibacteria*, *Bacteroidetes*, *Firmicutes*, *Actinobacteria,* and *Proteobacteria* (Figure 6).

## Discussion

Soil quality is important for plant establishment and growth (Shrestha et al., 2019). Selective planting in the Sb mining area significantly decreased the Zn concentration and increased Sb concentration, while other heavy metals (e.g., As, Cd, Pb, and Hg) showed no significant difference when compared to land abandonment. These results indicate that the effects of AFS and FFS on soil heavy metals were diverse. The vegetation of abandoned farmland is formed with natural succession, which is a form of self-repair for farmland and can lead to farmland restoration to some extent. There are many changes in plant compositions in the process of succession, and these plants have different absorption capacities for soil heavy metals. Our previous investigation found that some plants (e.g., *B. nivea*, *Pteridium aquilinum* var. *latiusculum*) have a high ability to absorb and transport the heavy metal Sb (Bai et al., 2021). The emergence of these plants with high Sb enrichment capacity during natural restoration may be the main reason for the obvious decline of Sb content in abandoned farmland. In addition, the investigation of Tang et al. (2020) on the regional distribution characteristics of heavy metals in Xikuangshan also showed that the contents of Sb in different land use were significantly different, among which the contents of ecological restoration grassland were relatively low. Interestingly, farmed farmland significantly reduces the content of the heavy metal Zn, which means that selective farming of maize and sweet potato may have the ability to absorb and transport Zn. Previous study also showed that crops (including maize and sweet potato) can absorb zinc in soil, and the absorption capacity at the root-soil interface is affected by root dry weight and morphology (e.g.,Ogiyama et al., 2010; Zhang et al., 2017). The application of farming fertilizer and soil microorganisms can affect the dry weight and morphology of roots in maize and sweet potato, and then change the accumulation of zinc in these crops (Ogiyama et al., 2010; Zhang et al., 2017).

Soil properties (e.g., heavy metals and soil physicochemical properties) are the main drivers of soil bacterial communities (Sibanda et al., 2019; Deng et al., 2020b; Duan et al., 2021; Khmelevtsova et al., 2022). Changes in soil properties caused by different agricultural land activities, such as abandonment and selective farming, are an important driving force for bacterial community dynamics. Our results observed that FFS increased the bacterial alpha diversity including Chao1 index, Simpson index, Shannon index and Pielou_−_e index and changed the beta diversity. These bacterial change might be caused by the farmland fertilization. Some studies documented that the farmland fertilization could increase bacterial alpha diversity and change bacterial beta diversity (Huang et al., 2019; Mei et al., 2021).

At the phylum level, *Proteobacteria* and *Acidobacteria* are the abundant phyla in both AFS and FFS, indicating that they have high survival and reproduction ability in the metal mining areas. This result is similar to other studies, showing these are the dominant phyla in Sb mines (Deng et al., 2020b; Duan et al., 2021), Zn mines (Luo et al., 2018), Cu mines (Sun et al., 2018), and gold mines (Sibanda et al., 2019). In this study, selective planting led to a significant decrease in the relative abundance of *Actinobacteria*, *Firmicutes*, and *Bacteroidetes*, and an increase in the relative abundance of *Chloroflexi*, *Acidobacteria*, *Gemmatimonadetes*, and *Rokubacteria*. A 40-year farmland fertilization experiment in Jilin Province, China also showed a significant increase in bacterial relative abundance of *Acidobacteria* and *Gemmatimonadetes* and a significant decrease in the *Actinobacteria*, *Bacteroidetes* and *Firmicutes* (Mei et al., 2021). In addition, our results also observed that *Actinobacteria* existed as a node phylum in the dominant seed network. The significant differences of this phylum caused by different farmland farming will further lead to the differences in the composition and abundance of other bacteria in the soil.

At the genus level, FFS significantly decreased the average abundances of *Bacillus*, *Rhodococcus*, *Sphingomonas*, and *67-14*, and increased the average abundances of *Haliangium*, *Acidothermus*, and *Rokubacteriales*. The abundance of different kinds of bacteria depends on the soil’s physical and chemical properties resulting from farming methods. For example, *Bacillus*, a bacterium with a strong tolerance for heavy metals, can effectively alleviate the toxicity of heavy metals for plants by biological accumulation and biotransformation (Aka and Babalola, 2016; Jan et al., 2019). Our previous research confirmed that *Bacillus* is more abundant at the beginning of succession with higher heavy metal pollution (Duan et al., 2021). *Sphingomonas* is favored in heavy metal contaminated soil and possess the ability to degrade pollutants including fungicides (Leys et al., 2004; Degrune et al., 2017). Studies have also confirmed that agricultural planting can significantly change the abundance of this genus (Babin et al., 2019; Chen et al., 2019). *Haliangium* has a higher abundance in the fertile soil of continuous cropping strawberries and mangoes (Li et al., 2019). Our previous research on rhizosphere bacteria in different restoration stages of the Sb mine confirmed that natural restoration would improve soil conditions and soil fertility, resulting in a significant increase in *Haliangium* content in the Sb mining area (Duan et al., 2021). So, we speculate that *Haliangium* may prefer soils with higher soil fertility. In addition, to our knowledge, there were no reports on the significant changes in the abundance of *Rhodococcus*, 67-14, *Acidothermus*, and *Rokubacteriales* caused by agricultural management, further tests were needed to clarify the potential link to agricultural management-driven soil bacterial properties.

Our experiments indicate that selective farming in the mining area not only changed the bacterial composition, but also significantly improved the soil bacterial richness, species evenness, and species diversity. The soil bacterial composition and diversity was affected by multiple environmental factors (e.g., Griffiths et al., 2011; Zeng et al., 2016). The main reasons for differences between planting methods may be the following:

1. Farming methods. The natural restoration of abandoned farmland polluted by heavy metals can lead to a change in bacterial community in the soil. The cultivation of agricultural soil polluted by heavy metals will also lead to a change in soil bacterial community composition. The research of Le Guillou et al. (2019) indicated that farming practices could effectively shape the richness and diversity of the soil bacterial community, and cultivation during rotation has an important impact on the formation of the bacterial community.
2. Heavy metals. Different concentrations of heavy metals can lead to various reactions of bacteria (Ippolito et al., 2010; Cavani et al., 2016). For example, Lejon et al. (2010) observed that Cu reduced the bacterial diversity in poor organic soil; on the contrary, Cu increased the bacterial diversity in soil rich in organic modifiers. The redundancy analysis from our experiment showed that Pb and Zn contents were closely related to the composition of the bacterial community in AFS, while the heavy metals Sb, As and Hg were closely related to the bacterial composition in FFS. In addition, studies have confirmed that heavy metals can form new complexes with organic matter, minerals, salts, and other components (Zhu et al., 2020). Forms of heavy metals also have various impacts on soil bacteria.
3. Soil fertility. The higher soil fertility of selective-farming farmland may improve bacterial diversity. Nitrogen availability is a limiting factor for bacterial activity in disturbed ecosystems (Raiesi, 2012). Large amounts of urea input into these N-poor soils may stimulate bacterial activity and respiration (Raiesi, 2004). Soil and crop management measures, including tillage and nitrogen fertilizer, can affect soil bacterial diversity by affecting the content and distribution of soil N, P, and K (García-Orenes et al., 2010; Castellano-Hinojosa et al., 2021; Khmelevtsova et al., 2022). Chao et al. (2016) and Santonja et al. (2018) confirmed that the composition of the bacterial community is mainly driven by soil nutrients at an abandoned mine site. Similarly, most of the communities were positively correlated with available N, P, and K in our study, which we held to be evidence that different farming methods indirectly regulated the composition and diversity of bacteria by changing soil factors.

## Conclusion

This study provides evidence to support the hypothesis that selective farming has a positive impact on measured soil quality indicators, compared to abandoned farmland. The abundances of *Haliangium*, *Acidothermus*, and *Rokubacteriales* in selective-farming farmlands significantly increased, while that of *Bacillus*, *Rhodococcus*, *Sphingomonas*, and *67-14* showed a significant decrease. The improvement of nutritional status and the increase in bacterial diversity in FFS showed that appropriate agricultural practice is conducive to the gradual restoration of cultivated land. Further investigation integrating bacterial functions and bacterial community analysis may be helpful to evaluate the impact of different agricultural practices on soil bacterial quality and function.

## Data availability statement

The datasets presented in this study can be found in online repositories. The names of the repository/repositories and accession number(s) can be found at: https://www.ncbi.nlm.nih.gov/, SAMN24619870, SAMN24619871, SAMN24619873, SAMN24619874, SAMN24619875, SAMN24619876, SAMN24619877, SAMN24619878, and SAMN24619879. The sequences from rhizosphere bacterial samples are available at NCBI SRA: BioProject PRJNA794295.

## Author contributions

RD conceived the research idea, conducted the field work and experiments, contributed to visualization, writing, and editing. YD, YZ, and YL conceived the research idea, writing, and editing. ZC contributed to sampling and conducted the statistical analysis. WH contributed to data curation and investigation. LY and GX conducted the field work and experiments. All authors contributed to the article and approved the submitted version.

## Funding

This work was supported by the Science and Technology Innovation Project of Hunan (2021NK2030, 2020NK2001, and 2021RC1016), the Graduate Innovation Project of Hunan Province (CX20211219), and the Agricultural Science and Technology Innovation Project of Hunan Province (2020cx84).

## Conflict of interest

The authors declare that the research was conducted in the absence of any commercial or financial relationships that could be construed as a potential conflict of interest.

## Publisher’s note

All claims expressed in this article are solely those of the authors and do not necessarily represent those of their affiliated organizations, or those of the publisher, the editors and the reviewers. Any product that may be evaluated in this article, or claim that may be made by its manufacturer, is not guaranteed or endorsed by the publisher.

## Supplementary materials

The Supplementary materials for this article can be found online at: https://www.frontiersin.org/articles/10.3389/fmicb.2022.953624/full#supplementary-material

Supplementary Figure S1 Site location of the study area.

Supplementary Figure S2 The seed network of dominant bacterial phyla.

## Abbreviations

AFS: Abandoned farmlands
FFS: Selective-farming farmlands
AN: Available N
AP: Available P
AK: Available K
PCoA: Principal coordinate analysis
RDA: Redundancy analysis
