# Supplementary figures and images for: Diversity and composition of soil bacteria between abandoned and selective-farming farmlands in an antimony mining area
Source: Front Microbiol. 2022 Jul 22;13:953624. doi: 10.3389/fmicb.2022.953624 (PMC9355163; doi:10.3389/fmicb.2022.953624)

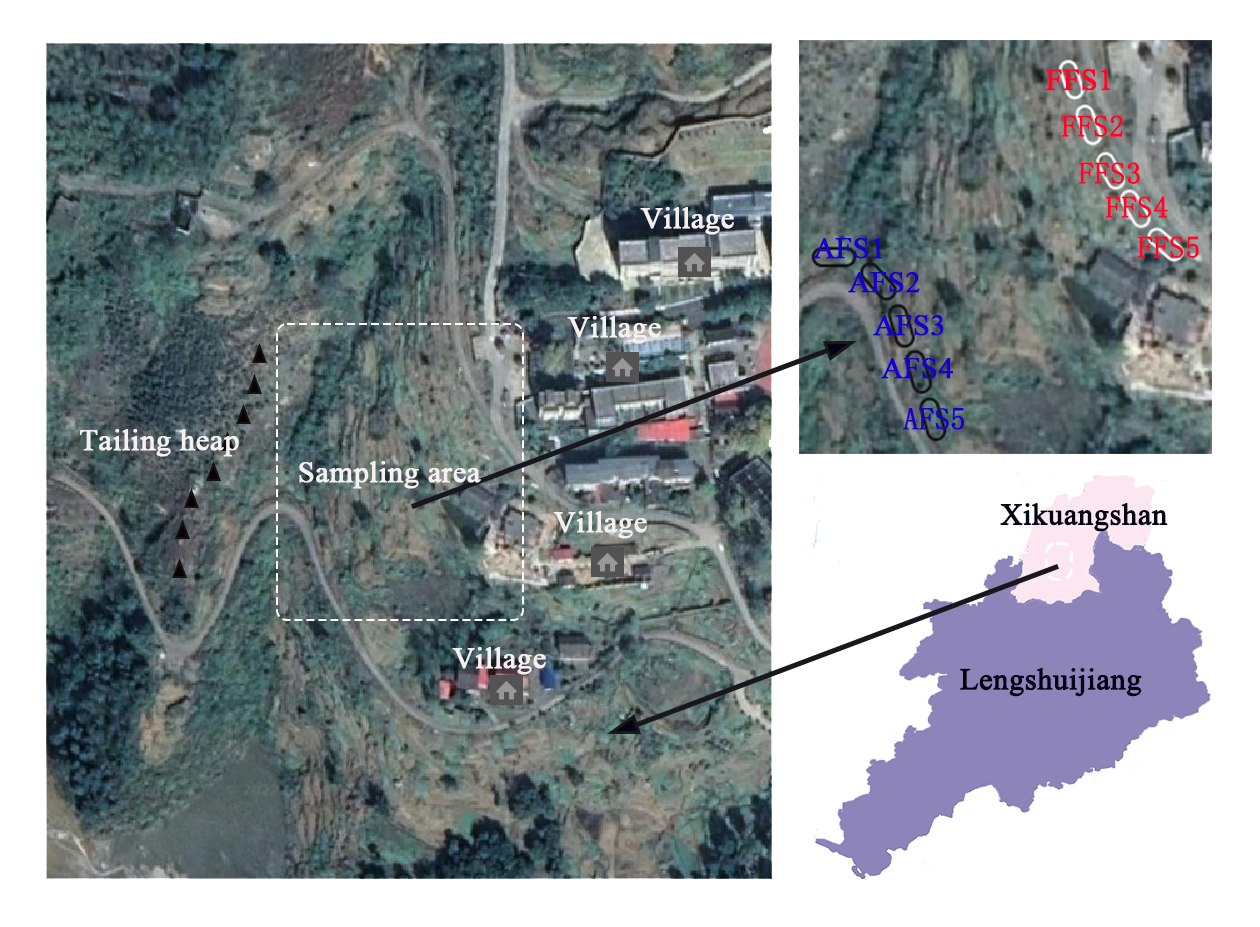

Supplement: Supplementary Figure S1 — Site location of the study area. [file Image_1.TIF]

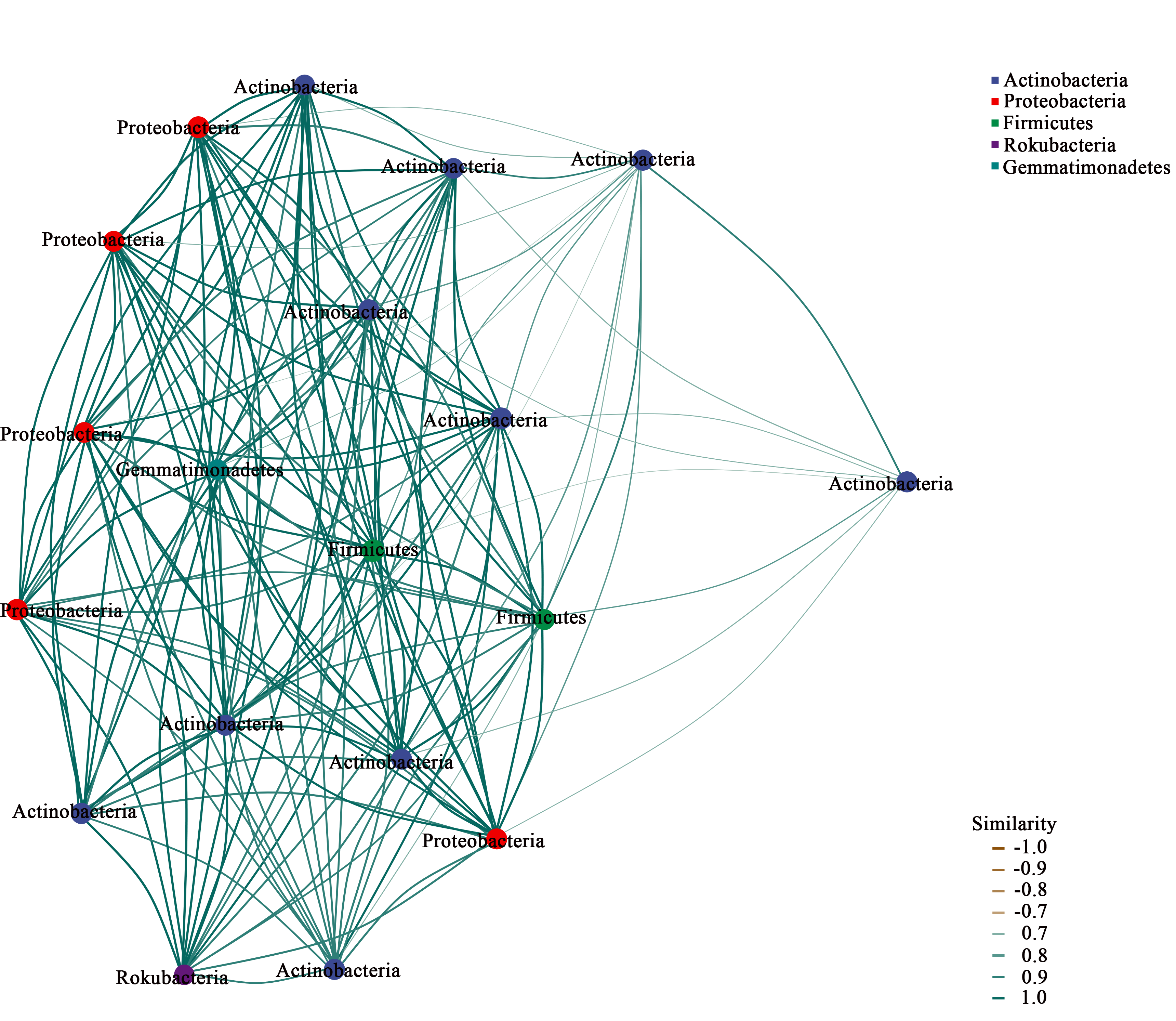

Supplement: Supplementary Figure S2 — The seed network of dominant bacterial phyla. [file Image_2.TIF]
